# miR-296-5p suppresses EMT of hepatocellular carcinoma via attenuating NRG1/ERBB2/ERBB3 signaling

**DOI:** 10.1186/s13046-018-0957-2

**Published:** 2018-11-29

**Authors:** Dong-Min Shi, Li-Xin Li, Xin-Yu Bian, Xue-Jiang Shi, Li-Li Lu, Hong-Xin Zhou, Ting-Jia Pan, Jian Zhou, Jia Fan, Wei-Zhong Wu

**Affiliations:** 1 Liver Cancer Institute, Zhongshan Hospital, Fudan University, Key Laboratory of Carcinogenesis and Cancer Invasion, Ministry of Education, 180 Fenglin Road, Shanghai, 200032 China; 2 Guang He Biao Guan Medical Technology (Beijing) Co.,LTD, Beijing, 101300 China; 3 Department of Chinese Traditional Medicine, ZhongShan Hospital, Fudan University, Shanghai, 200032 China

**Keywords:** HCC, miRNA, EMT, Metastasis

## Abstract

**Background:**

Accumulation of evidence indicates that miRNAs have crucial roles in the regulation of EMT-associated properties, such as proliferation, migration and invasion. However, the underlying molecular mechanisms are not entirely illustrated. Here, we investigated the role of miR-296-5p in hepatocellular carcinoma (HCC) progression.

**Methods:**

In vitro cell morphology, proliferation, migration and invasion were compared between HCC cell lines with up- or down-regulation of miR-296-5p. Immunofluorescence and Western blot immunofluorescence assays were used to detect the expression of EMT markers. Bioinformatics programs, luciferase reporter assay and rescue experiments were used to validate the downstream targets of miR-296-5p. Xenograft nude mouse models were established to observe tumor growth and metastasis. Immunohistochemical assays were conducted to study the relationships between miR-296-5p expression and Neuregulin-1 (NRG1)/EMT markers in human HCC samples and mice.

**Results:**

miR-296-5p was prominently downregulated in HCC tissues relative to adjacent normal liver tissues and associated with favorable prognosis. Overexpression of miR-296-5p inhibited EMT along with migration and invasion of HCC cells via suppressing NRG1/ERBB2/ERBB3/RAS/MAPK/Fra-2 signaling in vitro. More importantly, miR-296-5p disrupted intrahepatic and pulmonary metastasis in vivo. NRG1, as a direct target of miR-296-5p, mediates downstream biological responses. In HCC tissues from patients and mice, the levels of miR-296-5p and NRG1 also showed an inverse relationship.

**Conclusions:**

miR-296-5p inhibited EMT-related metastasis of HCC through NRG1/ERBB2/ERBB3/RAS/MAPK/Fra-2 signaling.

**Electronic supplementary material:**

The online version of this article (10.1186/s13046-018-0957-2) contains supplementary material, which is available to authorized users.

## Background

Hepatocellular carcinoma (HCC) treatment is a Gordian knot that still can not be untangled by the health professionals worldwide. Patients are often diagnosed at an advanced stage when intrahepatic and distant metastases have already occurred; moreover, surgical resection is only at best when treating an early stage HCC. Even though patients are qualified for receiving surgical resection, most are still doomed to postoperative recurrence and metastasis, resulting in a dismal survival [1–3]. Thus, to explore the metastatic mechanism in liver cancer is imperative.

The epithelial-mesenchymal transition (EMT) is a critical event in tumor metastasis [4]. During EMT process, tumor cells undergo a morphological transformation from epithelial to mesenchymal phenotype and simultaneously acquire enhanced invasive capabilities [5]. MicroRNAs (miRNAs) are a group of small noncoding single-stranded RNAs. They can act as tumor suppressors or promotors via the modulation of target gene expression at post-transcriptional levels in many human cancers [6]. Accumulation of studies reveals that miRNAs are involved in the EMT process. For instance, upregulation of miR-182 induced EMT and bone metastasis in breast cancer by targeting SMAD7 [7], and downregulation of miR-218 promoted EMT and lung cancer metastasis via Slug/ZEB2 signaling [8]. Our previous study also discovered the overexpression of miR-612 could reverse the EMT phenotype, attenuate cell growth and invasion by targeting AKT in HCC [9, 10]. These findings implicated that the aberrant expression profiles of specific miRNAs may lead to EMT progression in cancers.

At present, our institute establishes a series of monoclonal derivatives from one parental HCC cell line, which exhibits different metastasis potentials and tropisms in nude mice. Based on the ability of these cell lines and other commonly used HCC cell lines to metastasize in nude mice, we divided them into high and low metastatic groups. To identify a set of differentially expressed miRNAs that is relevant to metastasis in liver cancer, we used the whole transcriptome sequencing (WTS) analysis to screen the high and low metastatic group cell lines. With revisiting the fact that miR-296-5p was dysregulated between primary and lung metastatic foci in our previous study [9], miR-296-5p was the chosen candidate for studying its effects on HCC metastasis. Here, we investigated the role of miR-296-5p in HCC progression through functional experiments in vitro and orthotopic xenograft tumor model in vivo.

## Methods

### Cell description

MHCC97L, MHCC97H, HCCLM3, HCCLM1-S3, HCCLM1-S4, HCCLM1-S5, HCCLM1-S11, HCCLnM1-S11, HCCLnM1-S13 were established in our lab [11–13], CSQT-1, CSQT-2, SMMC-7721 were gifted from Second Military Medical University, Shanghai, China, Bel-7402, Bel-7404 were obtained from the Shanghai Cell Bank, Chinese Academy of Sciences. Other cells were purchased from the American Type Culture Collection. The HCC cell line Hep3B was cultured in RPMI-1640 medium with 10% fetal bovine serum (Hyclone, USA) and 1% penicillin-streptomycin (Invitrogen, USA). Other HCC cells were grown in DMEM medium (Hyclone, USA) with 10% fetal bovine serum and 1% penicillin-streptomycin. All cell lines were maintained in the incubator at 37 °C at 5% CO_2_. The metastatic status and evolutionary relationship of these cell lines were listed in Additional file 1: Table S1.

### miRNA sequencing

The total RNA samples were isolated from the HCC cell lines using the Ambion mirVana™ Total RNA Isolation Kit (Thermo Fisher Scientific, Carlsbad, CA, USA). Quality control was performed before library preparation using Illumina Truseq Small RNA library preparation kit V02 (Illumina San Diego, CA, USA). The validated DNA libraries were quantified using qPCR and then loaded on the Illumina HiSeq 2000 for miRNA sequencing at 100 bp single-end sequencing setting. The quality filtered sequencing reads were aligned to the human reference (Hg19) with Bowtie [14] and were further compared with the miRBase database (release 21) to identify known miRNAs. RPKM was calculated and normalized for each cell line, and the expression level of miRNA was then obtained. Hierarchical clustering of the miRNA expression profiles was performed using cluster [15] and visualized by Java Treeview [16]. Novel miRNAs were predicted by miRDeep2 [17]. DEseq was used for differential miRNA expression analysis between different groups of the cell lines. Any miRNA with FDR < 0.05 and fold change ≥1 was identified as differentially expressed miRNA.

### mRNA sequencing

The total RNA samples were extracted from HCC cell lines using the Ambion mirVana™ Total RNA Isolation Kit (Thermo Fisher Scientific). The quality control of the total RNA samples was performed before the RNA sequencing libraries were constructed using the Illumina TruSeq RNA Library Prep Kit V2. After the validation procession, the constructed libraries were then loaded on the Illumina HiSeq 2000 and ran at the setting of 50 bp paired-end reads.

The Illumina data pipelines including image analysis, base-calling and quality-filtering were performed using the Illumina sequence analysis software, Casava (version 1.8.2). All subsequent analyses were performed using the quality filtered sequencing reads. The reference genomes and the annotation files used in this study was downloaded from ENSEMBL database (http://www.ensembl.org/index.html). Bowtie 2 [14] was used for building the genome index, and clean data was mapped to the reference genome (hg19) using TopHat [18] with default parameters. Reads for each gene was counted by HTSeq [19], and RPKM (reads per kilobase of exon model per million mapped reads) was then calculated to measure the expression level of genes, the formula was defined as below:

$$ \mathrm{RPKM}=\frac{10^{\ast}\mathrm{R}}{\mathrm{NL}/{10}^3} $$

In which R was the number of reads uniquely mapped to the given gene; N was the number of reads uniquely mapped to all genes, and L was the total length of exons for the given gene. The RPKM approach could eliminate the effect of sequencing depth, and different gene lengths on gene expression measurement and the RPKM value was directly used for comparing the differences in gene expression among samples.

### Bioinformatics

The miRNA target prediction algorithms miRanda (http://www.microrna.org/microrna/home.do) and TargetScan (http://www.targetscan.org/) were used to predict the downstream targets of miRNAs.

### Cell transfections

NRG1 plasmid, small interfering RNA against NRG1 (siNRG1), against Fra-2 (siFra-2) and corresponding negative controls were purchased from GeneChem (GeneChem, Shanghai, China). These oligonucleotides and vectors were transfected into HCC cells using Lipofectamine 2000.

### Construction of stable cell lines

Ubi-Luc-MCS-IRES-Puromycin-miR-296-5p expression lentiviruses, mU6-MCS-Ubi-Luc miR-296-5p inhibitor lentiviruses and their corresponding negative controls were purchased from Shanghai GeneChem Co. The miR-296-5p expression lentiviruses were infected into MHCC97H cells and the inhibitor lentiviruses into Huh7 cells. Lenti-NRG1 viral particles were produced by cotransfection of 293 T cells with four plasmids: the pRSV-Rev packaging helper plasmid, pMDLg/pRRE, pMD2.G, and the pLenO-DCE Transfer Vector. Stably expressed clones were selected by qRT-PCR and immunoblotting assays after puromycin (1.5 μg/ml) treatment.

### Patient selection and TMA construction

Eighty-nine patients with primary HCC who underwent curative liver resection in Zhongshan Hospital (Shanghai, China) between July 2011 and April 2013 were enrolled. The follow-up information was updated till 1 January 2017. The paired HCC tissues and adjacent normal liver tissues from these 89 HCC patients were obtained and made into TMA according to the previously published method [20].

### Immunofluorescence

Cells scattered onto glass bottom dishes were incubated with E-cadherin antibody (1:200, Cell Signaling Technology, Danvers, MA, USA) and Vimentin antibody (1:200, CST) at 4 °C overnight after fixation in 4% paraformaldehyde and permeabilization in 0.2% Triton X-100. Then, the cells were incubated with Alexa Fluor 596-conjugated goat anti-rabbit-conjugated antibodies (1:200; ProteinTech Group, Shanghai, China) at room temperature for 1 h in the dark, followed by the counterstaining of cell nuclei using DAPI (Beyotime, Haimen, China) for 20 min. Images were observed under the Laser Scanning Confocal Microscope (Zeiss Germany, Oberkochen, Germany).

### Immunohistochemical (IHC) staining

Paraffin-fixed tumor tissues from xenograft model and tissue microarray (TMA) from 89 patients with paired HCC tissues and non-tumor tissues were deparaffinized, rehydrated and blocked with goat serum. Then, the slides were probed with primary antibodies against vimentin (1:200, CST), E-cadherin (1:200, CST) and NRG1 (1:200, ProteinTech) at 4 °C overnight, followed by the secondary antibody of goat anti-rabbit HRP conjugate (CST) at room temperature for 1.5 h. The 3′-diaminobenzidine solution (Beyotime, China) was used for brown color staining and hematoxylin (Beyotime, China) for counterstaining. The density of target proteins was measured with integrated optical density (IOD) by Image-ProPlus Version 6.2 software (Media Cybernetics Inc., Bethesda, MD) as previously described [21].

### Clone formation

Five hundred cells per well were seeded in 6-well plates and cultured for two weeks. Then, cells were stained with hematoxylin solution after washing three times with PBS. The numbers of visible colonies were calculated under a microscope.

### Animal models

Four-week-old male BALB/c nude mice purchased from Shanghai Laboratory Animal Co. Ltd. (Shanghai, China) was used for the establishment of HCC xenograft model [9]. Briefly, MHCC97H cells with different treatments along with their counterparts were implanted into the livers of nude mice. At the endpoint of 7 weeks, mice were sacrificed and tumor tissues were harvested, photographed, measured and examined by immunohistochemical staining. Tumor sizes were evaluated by the formula: Volume (mm^3^) = [width^2^ (mm^2^) × length (mm)]/2. Intrahepatic and pulmonary metastatic foci were examined by pathological identification.

### Luciferase reporter assay

The predicted binding sequences of NRG1 for miR-296-5p (wt-NRG1 3′-UTR) and the corresponding mutated sequence (mt-NRG1 3′-UTR) were cloned and inserted into the pmirGLO Dual-Luciferase miRNA Target Expression Vector (Promega, Madison, WI, USA). Wild-type constructs and mutants were transfected into HEK293T cells, together with miR-296-5p mimics or negative control in 24-well plates using Lipofectamine 2000 reagent (Thermo Fisher Scientific, Waltham, MA, USA). Forty-eight hours after transfection, luciferase activities were obtained using the Dual-Luciferase Reporter Assay System (Promega, Madison, WI, USA).

### Migration, invasion and wound-healing assay

Migration and invasion assays were performed by matrigel-uncoated and -coated transwell chambers (Corning, NY, USA). A density of 8 × 10^4^ cells per well was seeded in the upper chamber with serum-free DMEM. The lower chamber was filled with 500 μl DMEM medium supplemented with 10% fetal bovine serum (Hyclone, USA). Forty-eight hours later, migratory and invasive cells on the bottom surface of the filters were fixed in 4% paraformaldehyde, stained by 0.1% crystal violet solution (MedChem Express, Shanghai, China) and quantified under an inverted microscope (Olympus, Tokyo, Japan).

When cells seeded in 6-well plates were grown to approximately 80% confluence, a 10 μl sterile pipette tip was used to scratch across wound across the cell monolayer. The wounds were observed at 0, 24 and 48 h under an inverted microscope (Olympus, Tokyo, Japan). Three random fields were selected and measured. Migration index was calculated by the ratio of migrating area of treated cells to their counterparts.

### In situ hybridization

Tissue sections with 4 μm thickness on the TMA were incubated at 37 °C overnight. After deparaffinized in xylene, rehydrated with graded alcohol dilutes and digested with 8 mg/ml pepsin, slides were hybridized with 50 nm locked nucleic acid (LNA)-modified DIG-labeled probes for miR-296-5p (Exiqon, MA, USA) at 56 °C for 1 h. Then, slides were incubated in alkaline phosphatase conjugated anti-DIG Fab fragment solution at 4 °C overnight after stringency washes (5×, 1×, 0.2 × SSC) and 3% FBS block. Antibody signal was colorized with NBT and BCIP substrate (Roche, Mannheim, Germany) and the nuclei of cells were stained with Nuclear Fast Red (Sigma, MO, USA). The intensity of miRNA staining was measured with integrated optical density (IOD) by Image-ProPlus Version 6.2 software (Media Cybernetics Inc., Bethesda, MD).

### RNA isolation and quantification

Total RNAs were extracted from cultured cells with Trizol Reagent (Thermo Fisher Scientific, Waltham, MA, USA). The quality and integrity of RNAs were evaluated via A260/A280 ratio. For miRNA detection, first-strand DNA was synthesized from 2 μg of total RNA, and real-time PCR was performed in triplicate by the SYBR Green PCR method using an All-in-One miRNA qPCR Detection kit (GeneCopoeia, Rockville, MD, USA). The primers for has-miR-296-5p were purchased from GeneCopoeia (GeneCopoeia, Rockville, MD, USA). U6 was used as internal control. For mRNA detection, cDNA was prepared from 2 μg total RNA using a PrimeScript RT reagent kit (Takara Bio, Kyoto, Japan) and quantified in triplicate by using the SYBR Premix Ex Taq™ II (Takara Bio, Kyoto, Japan) with GAPDH as an internal control. Primers were listed in Additional file 2: Table S2. The threshold cycle (Ct) values were analyzed using the comparative Ct (-ΔCt) method.

### Western blot

Total proteins from cultured cells were lysed using RIPA buffer supplemented with protease and protease inhibitors (Roche, Mannheim, Germany). An equal amount of 50 μg proteins were fully electrophoresed on 6–10% SDS polyacrylamide gels and transferred to polyvinylidene difluoride membranes. The membranes were incubated with primary antibodies at 4 °C overnight after blocked with 5% non-fat milk at room temperature for 1 h. The primary antibodies against GAPDH were obtained from Abcam (Cambridge, MA, USA). The antibodies against E-cadherin, N-cadherin, Vimentin, Slug, Zeb1, NRG1, ERBB2, p-ERBB2, ERBB3, p-ERBB3, MEK1, p-MEK1, Erk, p-Erk, Raf, p-Raf, Fra-1, Fra-2, c-Fos, FosB, c-Jun and JunB (diluted at 1:1000 ratio) were purchased from Cell Signaling Technology (Beverly, MA, USA). Then, the membranes were incubated with goat anti-rabbit or anti-mouse secondary antibody (diluted at 1:5000 ratio) and then detected with ECL Detection System (Thermo Scientific, Rockford, IL, USA).

### Phalloidin staining

Phalloidin staining was performed to observe the cytoskeleton of MHCC97H cells stably overexpressed miR-296-5p, Huh7 cells with stably knockdown miR-296-5p, and their control counterparts. After fixed with 4% paraformaldehyde, permeabilized with 0.1% Triton× 100 and blocked with 1% BSA, cells were incubated in Alexa-488 conjugated phalloidin (Abcam) at room temperature for 1.5 h and photographed under the Laser Scanning Confocal Microscope (Zeiss Germany, Oberkochen, Germany).

### Ras activity assay

The relative activity of Ras GTPase was examined using a commercial Ras activation assay Kit (NewEast Biosciences, USA). Briefly, equal amounts of cell lysates (1.0 mg total protein) were sequentially incubated with anti-active RAS monoclonal antibody at 4 °C and protein A/G Agarose beads each for 30 min, then centrifuged at 5000×g for 1 min, washed with 0.5 ml 1 × Assay/Lysis buffer. Then the beads were boiled in protein loading buffer, and the released proteins were separated by 12% SDS-PAGE gels and transferred onto an Immobilon-P membrane (Millipore Corporation, Bedford, MA). RAS expression was queried using the anti-RAS antibody provided by the kit.

### Detection of secreted NRG1

Cells were normally cultured in DMEM media on a 10 cm dish and then repalced with unsupplemented DMEM/F12 growth media incubation overnight when grown to 80% confluency. The conditioned media were collected and centrifugated at 1500 rpm for 5 min and then at 4000×g for 40 min using Millipore Amicon Ultra (3 K) centrifugal filters. One-third the volume of 4 × Sample buffer was added to the concentrate in a 1.5 ml ultracentrifuge tube and heated at 95 °C for 5 min. A total of 20 μl of sample was immunoblotted using standard techniques and secreted NRG1 was detected using MAB377 monoclonal antibody from R&D Systems.

### Statistical analysis

Data were analyzed using SPSS 18.0 (Chicago, IL USA). The Student’s t-test was used to analyze differences between two groups, and two-way ANOVA was used when more than two groups were compared. The correlations between miR-296-5p and the clinical and pathological features were analyzed using the Spearman correlation test. Overall and disease-free survival curves were protracted using the Kaplan-Meier method and estimated by the log-rank test. Variables that were independently associated with overall survival (OS) and disease free survival (DFS) were identified using the Cox’s proportional hazards regression model. A two-tailed value of *P* < 0 .05 was considered significant.

## Result

### miR-296-5p inversely correlates with HCC aggressiveness

**Fig. 1:**
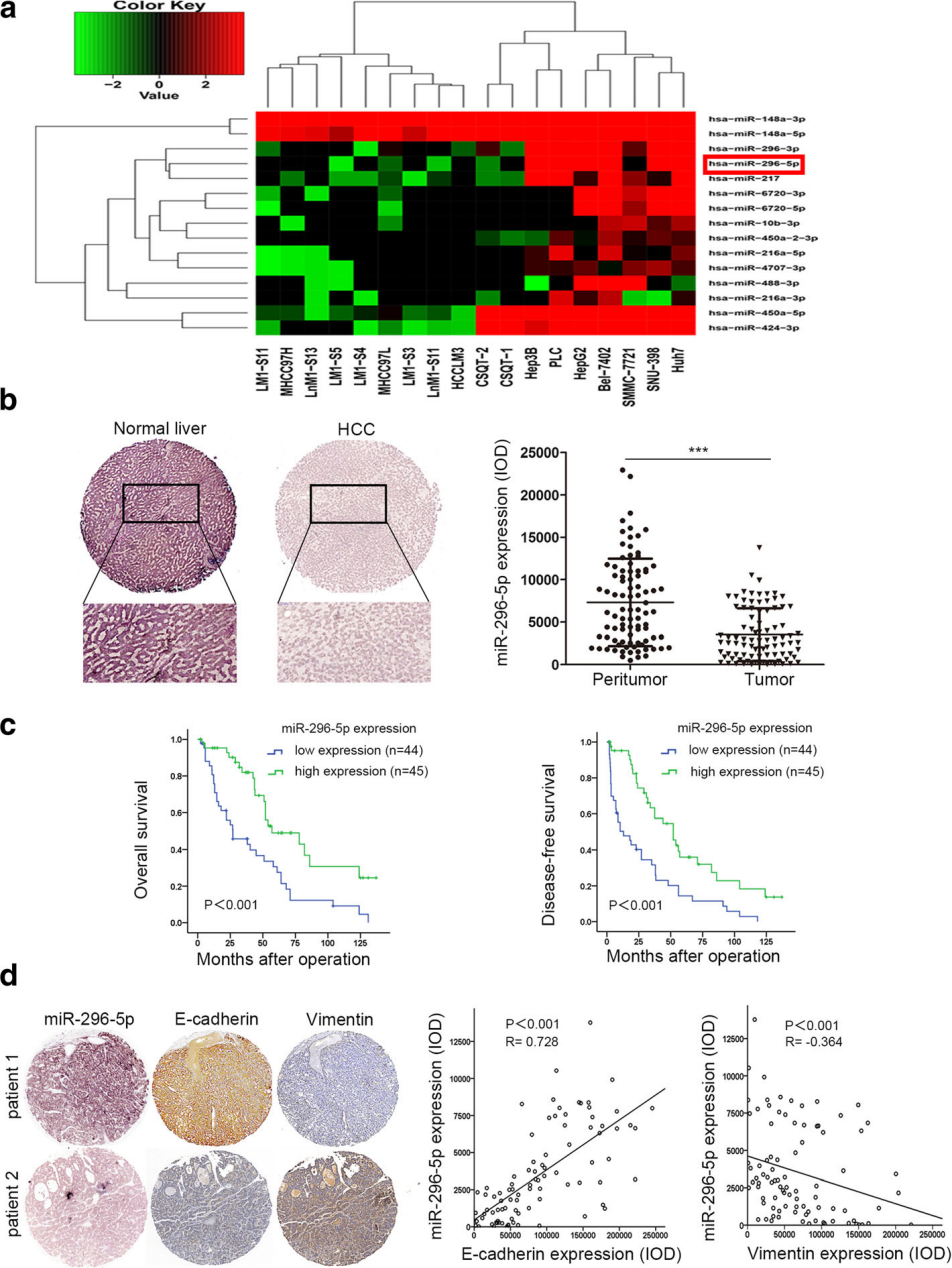
Endogenous levels of miR-296-5p inversely correlated with aggressive traits in HCC. (**a**) A heatmap of 15 differentially expressed miRNAs between HCC cell lines with high and low metastatic potentials. Columns represent 11 high and 7 low metastatic HCC cell lines. Colorgram depicts relative high (red) and low (blue) expression levels of miRNAs. (**b**) Representative images (left) and expression levels (right) of endogenous miR-296-5p in tumor tissues and paired adjacent normal liver tissues of 89 HCC patients by miRNA in situ hybridization. Magnification: 100× (above), 400× (below). (**c**) The OS and DFS in high and low miR-296-5p expressed HCC patients. (**d**) Representative images of the EMT-associated markers in HCC tissues with high or low level of miR-296-5p by IHC staining (left). Relationship between miR-296-5p levels and EMT-associated markers expression (right). magnification 100×. ***p < 0.001

To investigate the alterations of miRNA expression profiles during HCC metastasis, the WTS was carried out in 11 high and 7 low metastatic HCC cell lines using the Illumina HiSeq 2000 with an average sequencing depth of 4.75Gb per cell line. Of 1320 known and predicted human miRNAs, 15 miRNAs were differentially expressed between high and low metastatic groups (FDR < 0.05; fold change≥1) (Fig. 1a). Among them, miR-296-5p was once again identified to be negatively correlated with the metastatic potentials of HCC, which agreed with the result in our previous study [9]. To explore the clinical significance of miR-296-5p in HCC, in situ hybridization assay was performed in tumor and paired non-tumor liver tissues from 89 HCC patients. Apparently, miR-296-5p was mainly located in the cytoplasm, and its level was significantly downregulated in HCC tissues relative to adjacent normal liver tissues (3523 ± 327 vs 7325 ± 546, *P* < 0.001, Fig. 1b). Then, the patients were divided into miR-296-5p low-expressed (*n* = 44) and miR-296-5p high-expressed group (*n* = 45) when cut off with a median value of miR-296-5p expression. As shown in Additional file 3: Table S3, miR-296-5p was negatively associated with tumor size (*P* = 0.004), microvascular invasion (*P* < 0.001) and Edmondson-Steiner grade (*P* < 0.001). Higher OS (44 versus 23.5 months, *P* < 0.001) and DFS (34 versus 9.55 months, *P* < 0.001) were found in the miR-296-5p high-expressed group than the ones in low-expressed (Fig. 1c). The further Cox proportional hazards regression model was introduced. On univariate survival analysis, tumor size (*P* < 0.001), microvascular invasion (*P* < 0.001), Edmondson-Steiner grade (*P* = 0.002) and miR-296-5p expression (*P* = 0.001) reached significance for OS. Next, multivariate survival analysis on all parameters was performed. We found that OS time was significantly dependent on tumor size (*P* = 0.048), microvascular invasion (*P* = 0.032) and miR-296-5p expression levels (*P* = 0.017, Additional file 4: Table S4). For analysis of DFS time, tumor size ((P = 0.002), microvascular invasion (*P* < 0.001), Edmondson-Steiner grade (*P* < 0.001) and miR-296-5p expression (P < 0.001) reached significance in the univariate Cox proportional hazards regression model. However, only the microvascular invasion (*P* = 0.007), and miR-296-5p expression (*P* = 0.02, Additional file 5: Table S5) reached significance for DFS on multivariate survival analysis.

More strikingly, the miR-296-5p level in HCC tissues was positively correlated with E-cadherin expression (*R* = 0.728, P < 0.001, Fig. 1d), while negatively associated with Vimentin expression (*R* = − 0.364, *P* < 0.001, Fig. 1d). Besides, miR-296-5p was highly expressed in HCC cell lines with low metastatic capabilities (Additional file 6: Figure S1). These observations indicated that miR-296-5p could inhibit HCC progression and is a favorable diagnostic marker.

### miR-296-5p reverses EMT and inhibits cell proliferation, migration and invasion

**Fig. 2:**
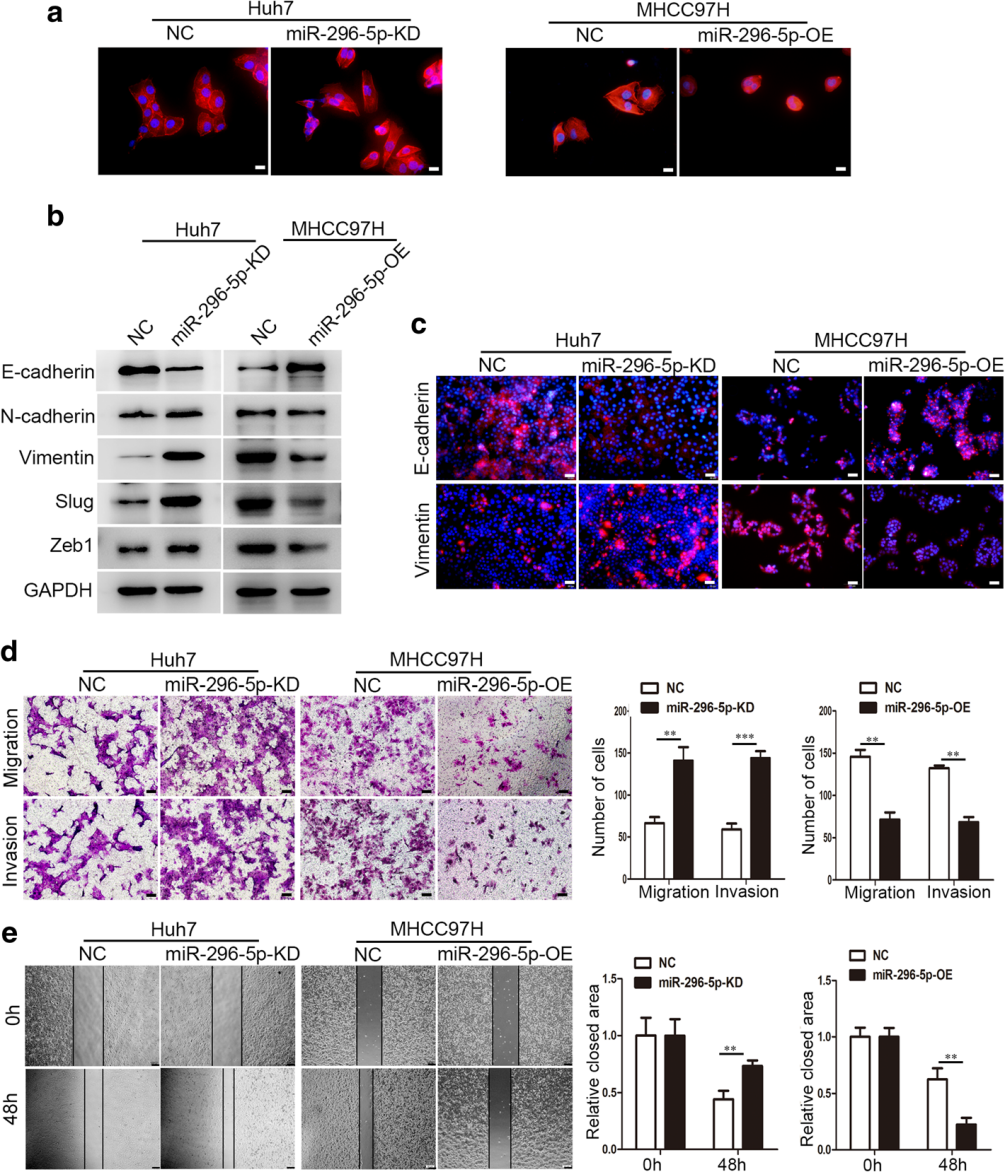
miR-296-5p inhibits EMT process and cell metastasis in vitro. (**a**) EMT-associated morphological changes in miR-296-5p-KD Huh7, miR-296-5p-OE MHCC97H cells and their negative control cells. Magnification 200×. (**b**) Western-blot analysis of protein levels of EMT markers and transcription factors in miR-296-5p-KD Huh7, miR-296-5p-OE MHCC97H cells and their negative control cells. GAPDH served as an internal control. (**c**) Immunofluorescence images of E-cadherin and Vimentin in miR-296-5p-KD Huh7, miR-296-5p-OE MHCC97H cells and their negative control cells. Magnification 200× (**d**) Transwell assays (left) and statistical results (right) of cellular migration and invasion at 48 h. magnification 100×. (**e**) Representative wound-healing images (left) and statistical results (right) of indicated cells at 0 and 48 h. magnification 100×. **p < 0.01, ***p < 0.001

To verify whether miR-296-5p displayed a specific role in HCC development, Huh7 and MHCC97H cells were used to construct miR-296-5p overexpressed (miR-296-5p-OE) and knockdown (miR-296-5p-KD) cell lines for further phenotype and function studies (Additional file 6: Figure S2). Intriguingly, compared with negative control cells, miR-296-5p-KD Huh7 cells had a mesenchymal phenotype like scattered spindles. Conversely, miR-296-5p-OE MHCC97H cells acquired an epithelial phenotype like condensed cobblestones (Fig. 2a). These phenomena suggested that miR-296-5p was involved in the EMT of HCC. Therefore, EMT-related markers and transcription factors were tested by Western blot and immunofluorescence assays. The protein levels of N-cadherin, Vimentin, Slug and Zeb1 were elevated, and E-cadherin expression was reduced in miR-296-5p-KD Huh7 cells, whereas the protein levels of N-cadherin, Vimentin, Slug and Zeb1 were reduced and E-cadherin expression was elevated in miR-296-5p-OE MHCC97H cells (Fig. 2b). Similar results were observed by immunofluorescence assay (Fig. 2c). The observations implied a suppressed role of miR-296-5p in the EMT of HCC.

Next, we assessed the influence of miR-296-5p on the migratory and invasive abilities of HCC since EMT is a key step in tumor metastasis [9]. The migratory and invasive capabilities of miR-296-5p-KD Huh7 cells were increased by more than 2.1- and 2.4- fold, respectively when compared with the control cells (*P* < 0.01, *P* < 0.001, Fig. 2d). In contrast, migration and invasion of miR-296-5p-OE MHCC97H cells decreased by more than 2.0- and 1.9-fold relative to their counterparts (*P* < 0.01, *P* < 0.01, Fig. 2d). The wound healing assay showed that miR-296-5p-KD Huh7 cells had a significantly faster wound closure than miR-296-5p-NC Huh7 cells (0.73 ± 0.05 vs. 0.44 ± 0.07, *P* < 0.01), whereas miR-296-5p-OE MHCC97H cells had remarkably lower wound closure than the control cells (0.22 ± 0.06 vs. 0.63 ± 0.10, *P* < 0.01; Fig. 2e). As excessive proliferation is an essential mechanism for cancer cells to metastasize [22], colony formation assays and CCK8 assays were then used to evaluate the effect of miR-296-5p on cell proliferation. Knockdown of miR-296-5p in Huh7 cells promoted cell proliferation; however, reconstitution of miR-296-5p in MHCC97H cells disrupted cell proliferation (Additional file 6: Figure S3a and b). Collectively, miR-296-5p attenuated EMT process and inhibited the proliferation, migration and invasion of HCC cells.

### NRG1 is a direct target of miR-296-5p

**Fig. 3:**
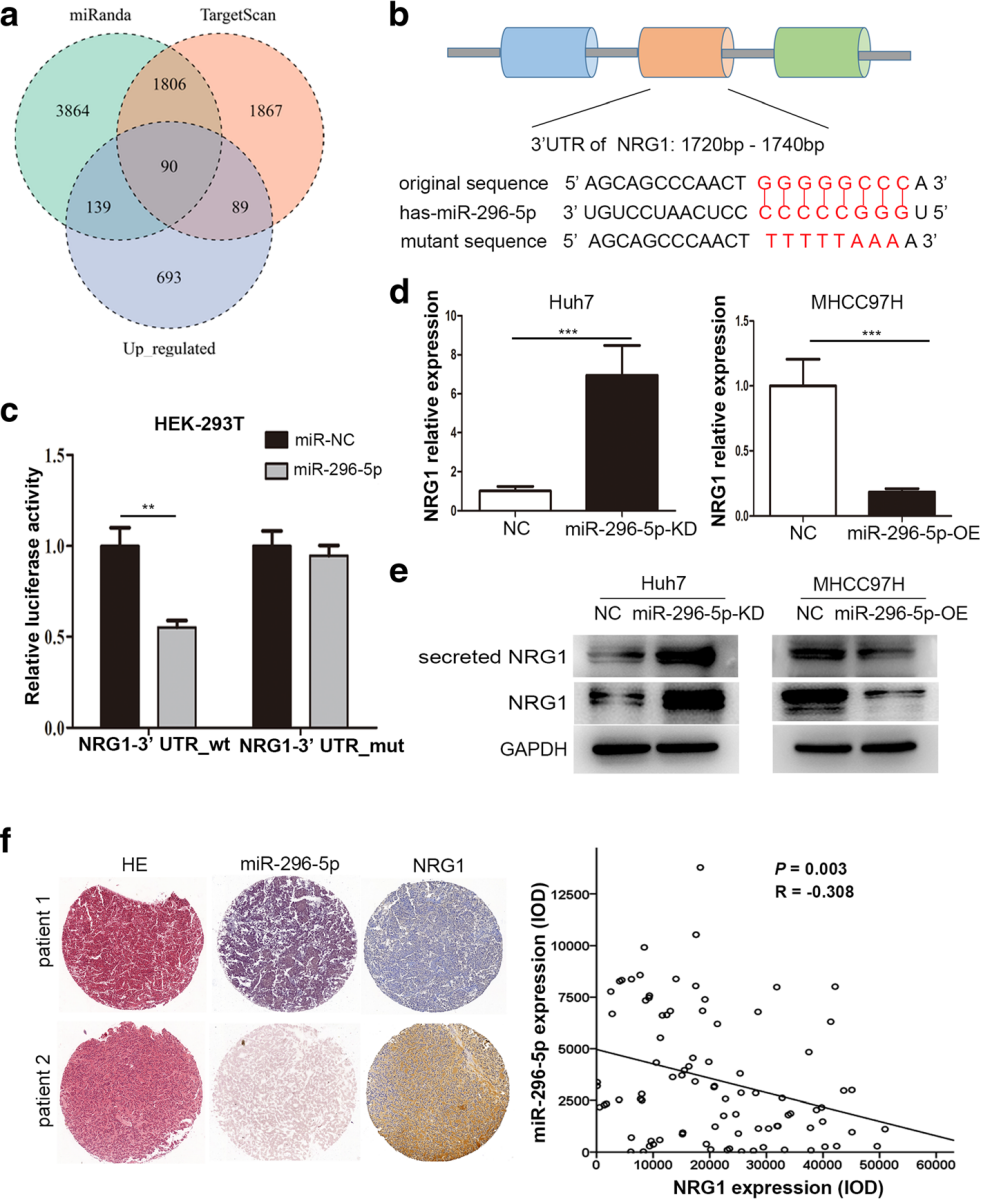
miR-296-5p directly targets NRG1. (**a**) Intersection of three profiles, including upregulated mRNAs in high metastatic cells compared with low metastatic cells, predicted targets of miR-296-5p separately by miRanda and TargetScan. (**b**) Schematic diagram of the dual luciferase miRNA target reporter vector, the wild type and mutant NRG1 3′-UTR sequences are shown with the miR-296-5p sequence. (**c**) Luciferase assays in HEK-293 T cells with wild-type or mutant NRG1 3′-UTR vectors and miR-296-5p mimic or NC. (**d**) qRT-PCR analysis of NRG1 mRNA levels in miR-296-5p-KD Huh7, miR-296-5p-OE MHCC97H cells and their negative control cells. (**e**) Western blot analysis of NRG1 and secreted NRG1 protein levels in miR-296-5p-KD Huh7, miR-296-5p-OE MHCC97H cells and their negative control cells. (**f**) Representative images of IHC staining of NRG1 in tumor tissues of HCC patients with high or low level of miR-296-5p (left), and an inverse correlation between the levels of NRG1 and miR-296-5p in HCC tissues (right). Magnification 100×. **p < 0.01, ***p < 0.001

To uncover miR-296-5p’s molecular mechanism underlying inhibitory effect on HCC EMT, we combined bioinformatics approaches with mRNA sequencing data to seek for the targets of miR-296-5p. The target candidates were selected based on these four criteria (1) predicted targets of miR-296-5p by miRanda and TargetScan, (2) upregulated mRNAs in high metastatic cells compared with low metastatic cells in our WTS analysis (Fig. 3a), (3) genes listed in the Cancer Gene Census (CGC, http://cancer.sanger.ac.uk), a public database, and (4) annotated in KEGG enrichment analysis (http://www.genome.jp/kegg/pathway.html); in the end, Neuregulin-1 (NRG1) was the selected candidate.

To verify whether NRG1 is the direct target of miR-296-5p, a 3’UTR element of NRG1 with wild-type (1720–1740 nts) or mutated sequences were designed, constructed and cloned into a dual luciferase reporter (Fig. 3b), then co-transfected with miR-296-5p mimics into HEK-293 T cells. The luciferase activities were significantly decreased by 45% in the reporter with wild-type binding sites, but not with mutant (*P* < 0.01, Fig. 3c), suggesting that miR-296-5p regulated NRG1 expression in a site-specific manner. Furthermore, the mRNA and protein levels of NRG1 in miR-296-5p-KD Huh7 cells were markedly elevated but reduced in miR-296-5p-OE MHCC97H cells when compared with their control counterparts (Fig. 3d and e). Besides, the protein level of secrected NRG1 from the conditioned media of miR-296-5p-KD Huh7 cells were also significantly higher than that of NC Huh7 cells, while lower from miR-296-5p-OE MHCC97H cells than NC MHCC97H cells (Fig. 3e). And a reverse relationship between miR-296-5p and NRG1 levels was observed in tumor tissues of HCC patients (*P* = 0.003, *R* = − 0.308, Fig. 3f). Taken together, NRG1 was a direct target of miR-296-5p.

### miR-296-5p inhibits tumor growth and metastasis in vivo

**Fig. 4:**
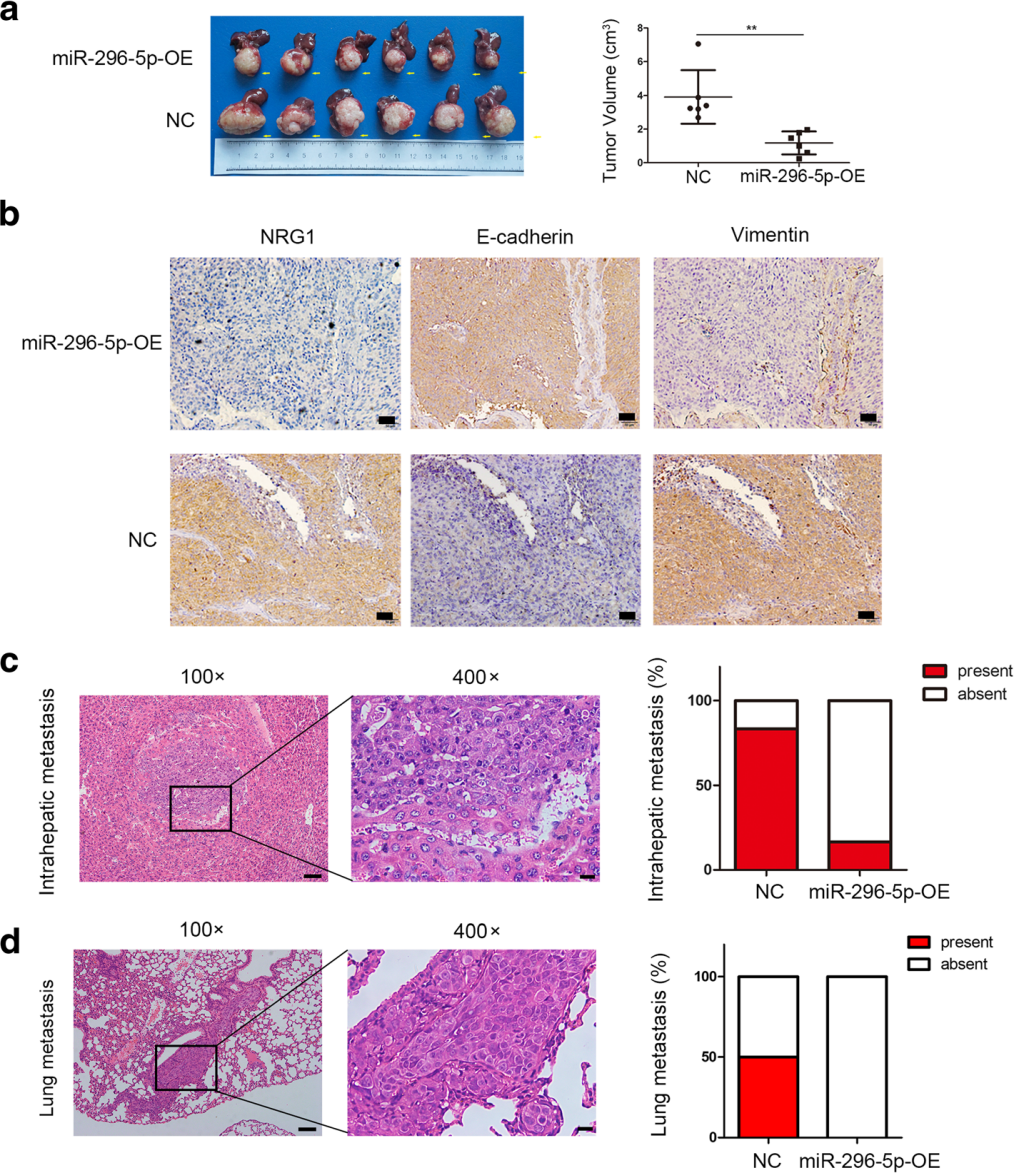
miR-296-5p inhibits tumor growth and lung metastasis in vivo. Xenograft tumor model was established in nude mice using miR-296-5p-OE MHCC97H cells and their negative control cells. (*n* = 6 for each group). (**a**) Images (left) and volumes (right) of primary liver tumors in recipient mice after 7 weeks. (**b**) The expression of NRG1, EMT markers (E-cadherin and Vimentin) in xenograft tumors with indicated cells analyzed by immunohistochemical analysis. Magnification 200×. (**c**) Hematoxylin and eosin (H&E) staining of metastatic liver nodules (left) and the percentage of mice with or without metastatic nodules in the livers (right). (**d**) Representative pictures for lung metastasis (left) and the percentage of mice with or without metastatic nodules in the lungs (right). magnification × 100 (left); 400× (right). **p < 0.01

Next, an orthotopic xenograft tumor model was used to explore the effects of miR-296-5p on tumor growth and metastasis in vivo. After 7 weeks, miR-296-5p-OE MHCC97H group exhibited smaller tumors than NC group (volume, 1.18 ± 0.28 vs 3.91 ± 0.65 cm^3^, *P* < 0.01, Fig. 4a). Consistent with in vitro result, miR-296-5p-OE MHCC97H xenograft tumors expressed lower levels of NRG1 and Vimentin but higher E-cadherin relative to NC group when analyzed by IHC staining (Fig. 4b). Meanwhile, the rates for intrahepatic and pulmonary metastasis of HCC cells were checked. As shown in Fig. 4c and d, the intrahepatic and lung metastasis rate of NC group was 83% (5/6) and 50% (3/6) respectively, whereas 17% (1/6) intrahepatic metastasis and no lung metastasis (0/6) were found in miR-296-5p-OE MHCC97H group. All these results suggested that ectopic forced expressions of miR-296-5p conspicuously suppressed HCC tumor growth and metastasis in vivo system.

### NRG1 positively regulates HCC EMT and metastasis

**Fig. 5:**
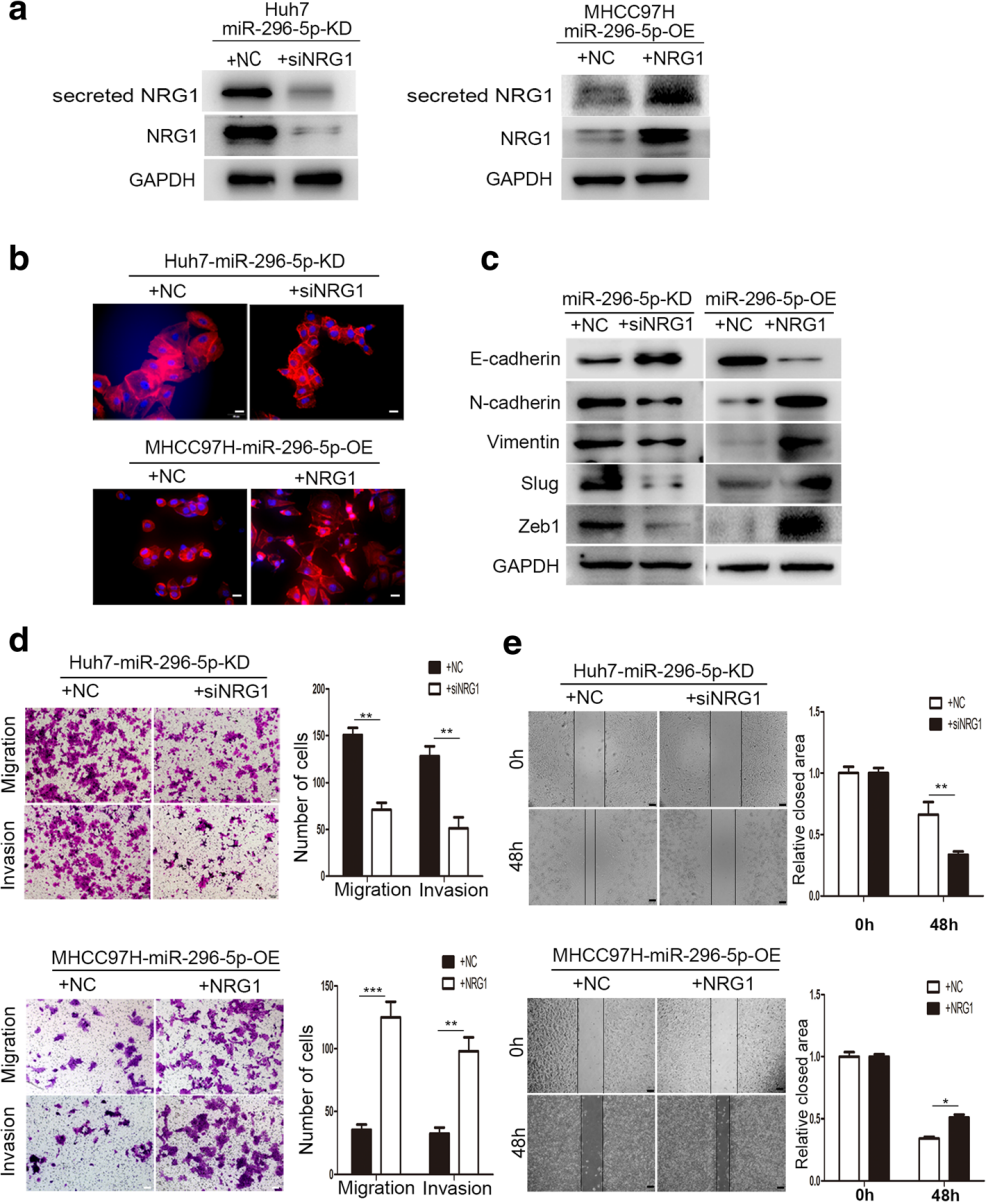
miR-296-5p suppresses EMT and metastasis through NRG1. The miR-296-5p-KD Huh7 cells were transfected with siNRG1 and control siRNA, the miR-296-5p-OE MHCC97H cells were transfected with NRG1-expressing plasmids or the control plasmids for 48 h. The NRG1, secreted NRG1 proteins levels (**a**), EMT-associated morphological changes (**b**), EMT markers (E-cadherin and Vimentin) (**c**), the cellular metastasis (**d**) and motion ability (**e**) in the indicated cells. Magnification 200×. *p < 0.05, **p < 0.01, ***p < 0.001

To investigate whether miR-296-5p attenuated EMT and metastasis by NRG1, we examined the relationship between NRG1 and miR-296-5p-mediated EMT. Firstly, the siNRG1 and NRG1 plasmid were respectively transfected into miR-296-5p-KD Huh7 and miR-296-5p-OE MHCC97H cells, and the transfection efficiency was measured by western blots (Fig. 5a). As shown in Fig. 5b, knockdown of NRG1 in miR-296-5p-KD Huh7 cells abrogated EMT phenotype, followed by upregulated expression of E-cadherin and downregulation of N-cadherin, Vimentin, Slug, and Zeb1 (Fig. 5c). On the contrary, through overexpression of NRG1 in miR-296-5p-OE MHCC97H cells, epithelial-like cells returned to mesenchymal-like phenotype (Fig. 5b), as evidenced by elevated N-cadherin, Vimentin, Slug, and Zeb1 and reduced E-cadherin expression (Fig. 5c). Similarly, cell migration and invasion capabilities were significantly inhibited in siNRG1-transfected miR-296-5p-KD Huh7 cells by more than 2.1- and 2.5-fold (*P* < 0.01, *P* < 0.01, Fig. 5d); moreover, prominently promoted in NRG1 plasmid-transfected miR-296-5p-OE MHCC97H cells by more than 3.5- and 3.0-fold, when compared with their control counterparts (*P* < 0.001, *P* < 0.01, Fig. 5d). Wound healing assay further verified these results (Fig. 5e). In vivo HCC xenograft tumors model also showed that overexpression of NRG1 could reverse the decreased rates of intrahepatic and pulmonary metastasis caused by miR-296-5p overexpression (Additional file 7: Figure S4a and b). These findings suggest that miR-296-5p inhibits EMT and metastasis of HCC cells by suppression of NRG1.

### ERBB2/ERBB3/RAS/MAPK signaling is critical for the biological behavior of miR-296-5p in HCC

**Fig. 6:**
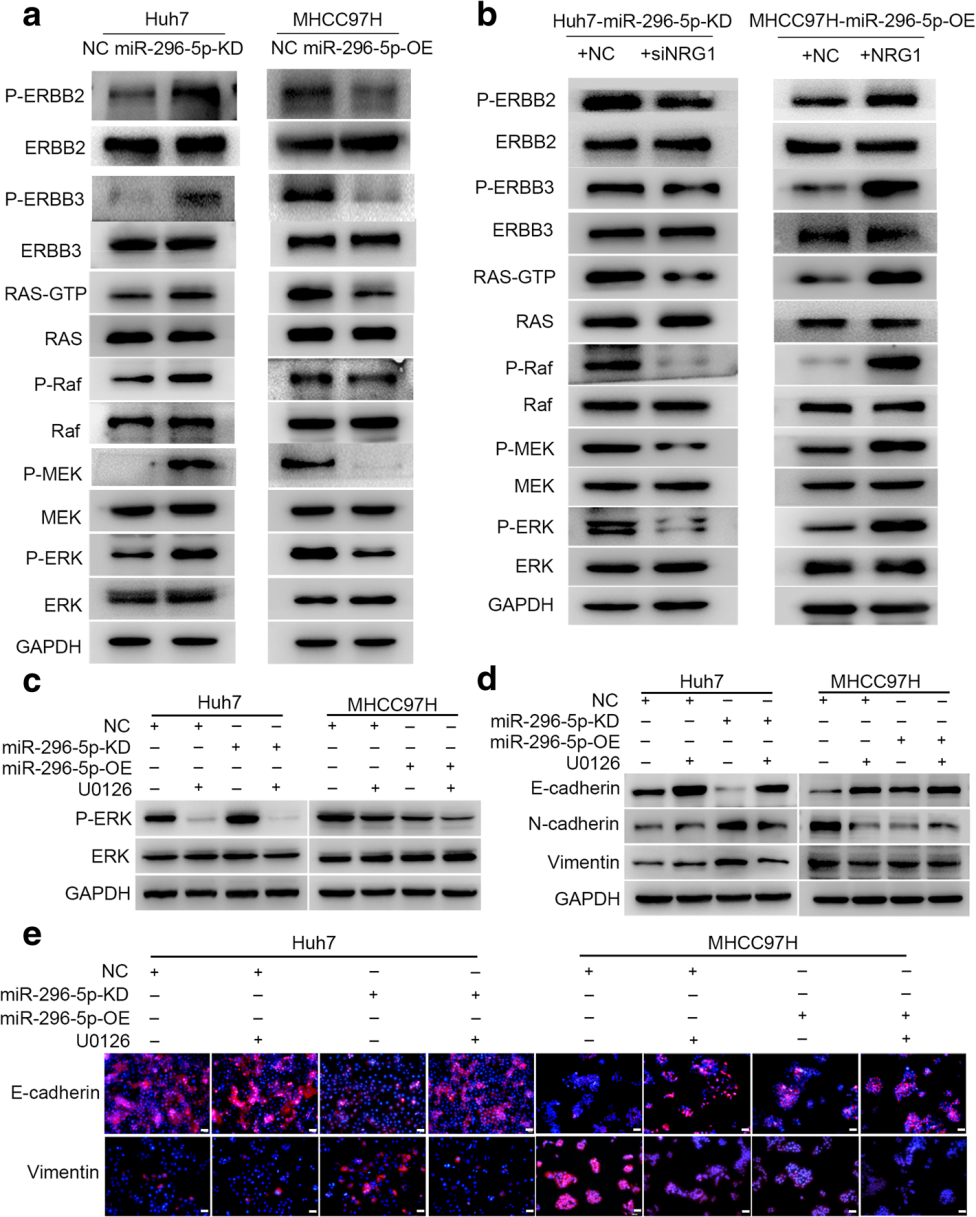
miR-296-5p mediates HCC EMT by ERBB2/ERBB3/RAS/MAPK signaling. (**a**) The levels of ERBB2, ERBB3 and RAS/MAPK signaling-related proteins in miR-296-5p-KD Huh7, miR-296-5p-OE MHCC97H cells and their corresponding control cells. (**b**) The protein levels of ERBB2, ERBB3 and RAS/MAPK signaling-related molecules in siNRG1-transfected miR-296-5p-KD Huh7 cells, NRG1-expressing plasmids transfected miR-296-5p-OE MHCC97H cells and their counterparts. The protein expressions of P-ERK (**c**) and EMT markers (**d** and **e**) in miR-296-5p-KD Huh7, miR-296-5p-OE MHCC97H cells and their corresponding control cells after U0126 treatment

The ERBB family members (ERBB2, ERBB3, and ERBB4) are the dominant receptors for NRG1 and this ligand-receptor interaction can activate a series of downstream signal cascades, such as Janus Kinase (JAK)/signal transducer and (STAT) transcription activator, phosphatidylinositol 3′-kinase (PI3K)-Akt and mitogen-activated protein kinase (MAPK), resulting in cell proliferation, invasion, and angiogenesis [23]. Since the distinct mechanisms through which NRG1 exert functions mostly depend on the types of tissues or organs; thus, we next tried to explore the downstream pathways underlying the EMT and metastasis mediated by NRG1 in HCC cells by western blot. The protein levels of P-ERBB2/3, RAS-GTP, P-Raf, P-MEK, and P-ERK were significantly upregulated in miR-296-5p-KD Huh7 cells. Conversely, these molecules were downregulated in miR-296-5p-OE MHCC97H cells, compared with their control cells (Fig. 6a). These suggested an inactivation of MAPK pathway induced by miR-296-5p overexpression.

Furthermore, the expressions of P-ERBB2/3, RAS-GTP, P-RAF, P-MEK, and P-ERK were decreased in siNRG1-transfected miR-296-5p-KD Huh7 cells and were increased in NRG1 plasmid-transfected miR-296-5p-OE MHCC97H cells (Fig. 6b). To test whether MAPK signaling was involved in the miR-296-5p-mediated EMT and metastasis of HCC cells, U0126 (an inhibitor of MEK/ERK signaling) was employed. The P-ERK levels were significantly reduced in miR-296-5p-KD Huh7 and miR-296-5p-NC MHCC97H cells with U0126 treatment, relative to their counterpart cells (Fig. 6c), followed with elevated E-cadherin and reduced N-cadherin, Vimentin expression by Western Blot and immunofluorescence assay (Fig. 6d and e). Besides, the invasion and migration capabilities were further inhibited in miR-296-5p overexpressed cells (miR-296-5p-NC Huh7 or miR-296-5p-OE MHCC97H) than low miR-296-5p expressed ones (miR-296-5p-KD Huh7 or miR-296-5p-NC MHCC97H) when combined with U0126 (Additional file 7: Figure S5a and b). These results indicate that inactivation of NRG1/ERBB2/ERBB3/RAS/MAPK signaling is critical for the biological behavior of miR-296-5p in HCC.

### Fra-2 is a key transcription factor for miR-296-5p-mediated HCC EMT

**Fig. 7:**
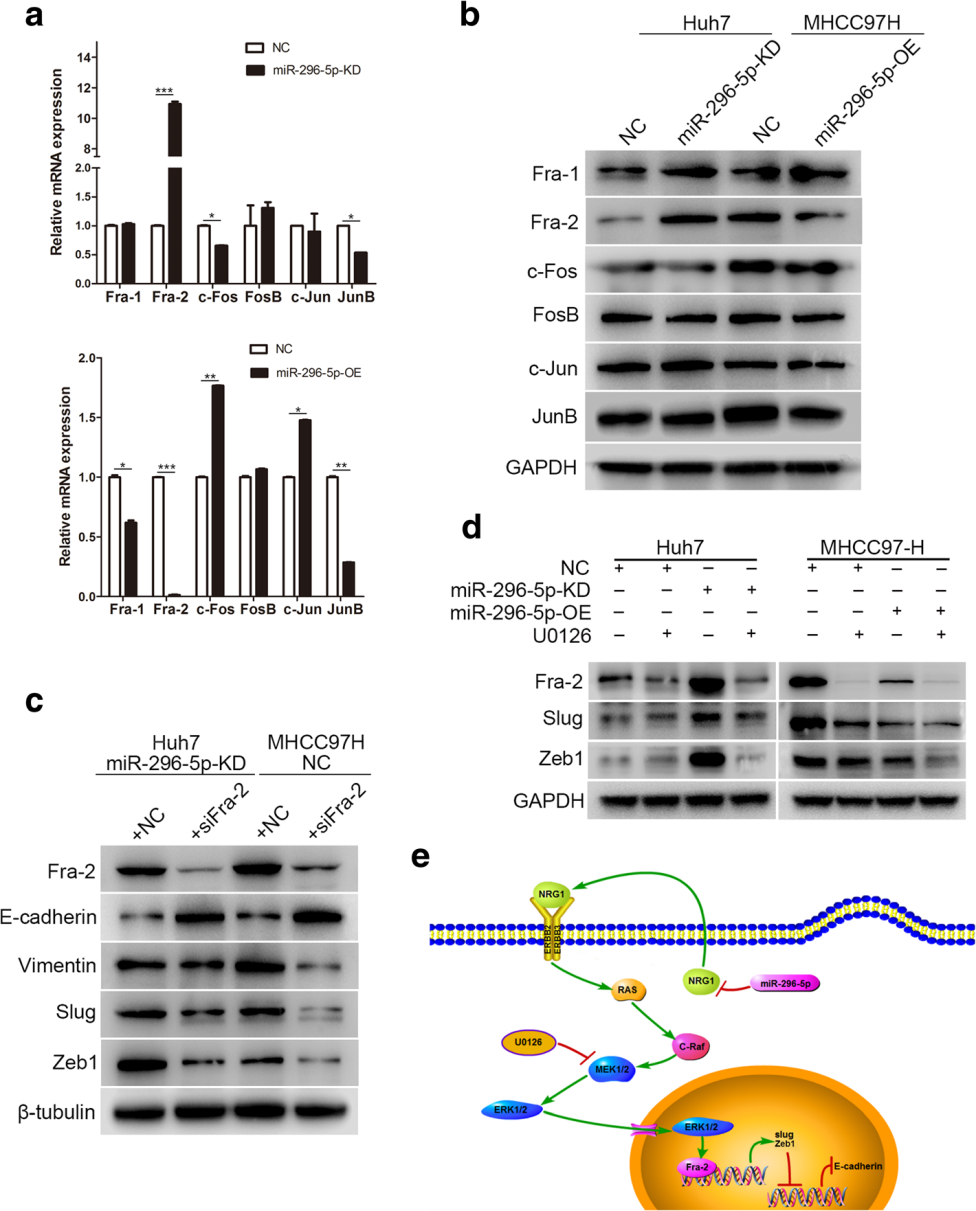
Fra-2 is a key transcription factor for miR-296-5p-mediated HCC EMT. The mRNA (**a**) and protein levels (**b**) of AP-1 transcription factors in miR-296-5p-KD Huh7, miR-296-5p-OE MHCC97H cells and their negative control cells. (**c**) The changes of EMT-related molecules in miR-296-5p-KD Huh7 and miR-296-5p-NC MHCC97H cells after Fra-2 knockdown. (**d**) The protein levels of Fra-2, Slug and Zeb1 after U0126 incubation. (**e**) Schematic diagram of NRG1/ERBB /RAS/MAPK/Fra-2 axis inactivation and HCC EMT reversal induced by miR-296-5p overexpression. *p < 0.05, **p < 0.01, ***p < 0.001

The previous study has reported that the activated ERK can translocate into nuclei to activate transcription factors like Jun and Fos. Jun (such as c-Jun and Jun B) dimerize with Fos (c-Fos, FosB, Fra-1, and Fra-2) proteins to compose activator protein-1 (AP-1), which binds to TRE/AP-1 elements and then activates transcription [24]. Thus, to verify which is the direct effect factor of ERK in miR-296-5p-mediated EMT and metastasis of HCC cells, the mRNA and protein levels of c-Jun, JunB, c-Fos, FosB, Fra-1 and Fra-2 were measured. Intriguingly, only Fra-2 mRNA and protein levels were markedly higher in miR-296-5p-KD Huh7 and miR-296-5p-NC MHCC97H cells when compared with their counterpart cells (Fig. 7a and b). Along with the observation that Slug and Zeb1 levels were markedly increased in low miR-296-5p expressed cells (miR-296-5p-KD Huh7 or miR-296-5p-NC MHCC97H), we preliminarily postulated that Fra-2 might modulate Slug and Zeb1 expression. Through the knockdown of Fra-2 with siRNA (siFra-2), the protein levels of Vimentin, Slug and Zeb1 were dramatically reduced, and cadherin expression was elevated (Fig. 7c). Besides, Fra-2, Slug and Zeb1 levels were significantly decreased after U0126 treatment (Fig. 7d). Above all, miR-296-5p attenuated EMT by inhibiting the NRG1/ERBB2/ERBB3/RAS/MAPK/Fra-2 signaling in HCC cells (Fig. 7e).

## Discussion

Ample evidences have demonstrated that miRNAs, besides of classic protein coding genes, are the major drivers on HCC metastasis at the post-transcriptional levels [25, 26]. Therefore, identification of metastasis-related miRNAs is a critical step for understanding the molecular mechanisms underlying HCC progression. Up to date, research studies have reported the involvement of miR-296-5p in several types of cancer’s occurrences. The distinct functions which miR-296-5p exerts mostly depend on the types of tissues or organs. For example, it acts as an oncogene in gastric cancer and glioblastomas [27–29]; whereas it displays a tumor-suppressive role in prostate cancer and lung cancer [30, 31]. Moreover, miR-296-5p contributes to radiotherapy resistance in laryngeal carcinoma [32]. Our previous studies found that miR-296-5p was down-regulated in metastatic lung foci versus their primary tumor tissues by microarray analysis [9]. In the present study, miR-296-5p was significantly down-regulated in high metastatic HCC cell lines with WTS analysis, which hints that miR-296-5p may exert a key role in HCC progression. Here, we first demonstrated that miR-296-5p inhibited the EMT process by regulating NRG1 expression in HCC probably through a cell-autonomous mechanism.

Tumor metastasis is a complex process that involves a series of events [33]. During the invasive-metastatic cascade, EMT could endow noninvasive tumor cells with metastasis potentials, enable invasive cells to migrate in the extracellular matrix, crossover basal membrane of vessels, survive in the bloodstream and initiate a metastatic clonal growth on the second organ with the help of angiogenesis [34]. Moreover, EMT also helps promote hepatic fibrosis by providing a pre-metastatic microenvironment [35]. A plethora of studies confirms the influential role of miRNAs in EMT process [36, 37]. In the current study, miR-296-5p showed a positive correlation with E-cadherin level, and both in vitro and in vivo experiments also demonstrated that miR-296-5p could reverse the EMT phenotype and metastatic capability in HCC cells. NRG1, a member of NRG family, is known as a key growth factor for the development of the normal nervous system and the occurrence of schizophrenia, which is also involved in cancer progression [38, 39]. The relationship between miR-296-5p and NRG1 in HCC was further clarified here. More importantly, the effects of miR-296-5p modulation on EMT in HCC could be reversed through the knockdown of NRG1 by the siRNA. The data support that miR-296-5p could inhibit EMT by suppressing the expression of NRG1 and subsequent secreted NRG1.

Previous studies reported that NRG1 exerts its effects in cancers by directly binding to ERBB3 or ERBB4 which heterodimerizes with ERBB2 and the ligand-receptor interaction, causes the phosphorylation of receptors and activation of signaling cascades [40]. Our study showed a markedly elevated level of phosphorylated ERBB2 and ERBB3 while no obvious difference found in ERBB4, thus suggested the heterodimer of ERBB2/ERBB3 might be the dominant receptors for NRG1 in HCC cells. And then RAS/MAPK/Fra-2 signaling was further identified as the downstream pathway underlying the NRG1/ERBB2/ERBB3 signaling in HCC cells. Of all, Fra-2 was the necessary mediator from ERK to EMT-associated transcription factors (Slug and Zeb1). Inhibition of NRG1, ERK or Fra-2 could attenuate the EMT process induced by the miR-296-5p knockdown in HCC cells. Hence, miR-296-5p attenuated EMT by inhibiting NRG1 and ERBB2/ERBB3/ERK/Fra-2 axis in HCC cells. In conclusion, a novel insight of miR-296-5p on HCC EMT was identified in the study, and miR-296-5p might potentially be an effective biomarker for HCC progression. However, further clinical trials in a multicenter are need.

## Conclusions

In conclusion, the findings on the antimetastasis effect of miR-296-5p in HCC cells provide a clue for treating an advanced stage of HCC and deserve attention in the clinical practice of precision medicine.

## Additional files

Additional file 1: **Table S1.** HCC cell lines with different metastatic potentials. (DOCX 20 kb)

Additional file 2: **Table S2.** Sequence of primers for Real-time PCR. (DOC 34 kb)

Additional file 3: **Table S3.** Correlations of miR-296-5p expression with the clinicopathological features of HCC. (DOCX 18 kb)

Additional file 4: **Table S4.** Univariate and multivariate analyses of risk factors associated with overall survival of HCC patients. (DOCX 20 kb)

Additional file 5: **Table S5.** Univariate and multivariate analyses of risk factors associated with disease-free survival of HCC patients. (DOCX 20 kb)

Additional file 6: **Figure S1.** The endogenous levels of miR-296-5p in HCCLM3, MHCC97H, MHCC97L, Huh7 and HepG2 cell lines quantified by qRT-PCR analysis. U6 was used as a control. **Figure S2.** The miR-296-5p level in Huh7 cells (left) transfected with miR-296-5p knockdown lentiviral vector and MHCC97H cells (right) transfected with miR-296-5p overexpression lentiviral vector analyzed by qRT-PCR. **P < 0.01. **Figure S3.** The effect of miR-296-5p on HCC cell proliferation. Cell growth capabilities in miR-296-5p-KD Huh7, miR-296-5p-OE MHCC97H cells and their corresponding controls by colony formation (a) and CCK8 assays (b). **P < 0.01. (TIF 621 kb)

Additional file 7: **Figure S4.** miR-296-5p suppresses in vivo metastasis through NRG1. (a) Hematoxylin and eosin (H&E) staining of metastatic liver nodules (left) and the percentage of mice with or without metastatic nodules in the livers (right). (b) Representative pictures for lung metastasis (left) and the percentage of mice with or without metastatic nodules in the lungs (right). magnification × 100 (left); 400× (right). **Figure S5.** miR-296-5p mediates HCC cell metastasis through MAPK signaling. (a and b) The cellular invasive and migratory capability in miR-296-5p-KD Huh7, miR-296-5p-OE MHCC97H cells and their corresponding control cells after U0126 treatment. *p < 0.05, **p < 0.01, ***p < 0.001. (TIF 4105 kb)

## Abbreviations

AP-1: Activator protein-1
DFS: Disease-free survival
EMT: Epithelial-mesenchymal transition
FDR: False discovery rate
HCC: Hepatocellular carcinoma
IHC staining: Immunohistochemical staining
JAK/STAT: Janus kinase/signal transducer and activator of transcription
MAPK: mitogen-activated protein kinase
miRNAs: microRNAs
mRNA: messenger RNA
NRG1: Neuregulin-1
OS: Overall survival
PI3K/Akt: Phosphatidylinositol 3′-kinase/AKT
siRNA: Small interfering RNA
TMA: Tissue microarray
WTS: Whole transcriptome sequencing

## References

[1] Tang ZY, Ye SL, Liu YK, Qin LX, Sun HC, Ye QH, Wang L (2004). A decade's studies on metastasis of hepatocellular carcinoma. J Cancer Res Clin Oncol.

[2] Desai JR, Ochoa S, Prins PA, He AR (2017). Systemic therapy for advanced hepatocellular carcinoma: an update. J Gastrointest Oncol.

[3] Finn Richard S., Zhu Andrew X., Farah Wigdan, Almasri Jehad, Zaiem Feras, Prokop Larry J., Murad Mohammad Hassan, Mohammed Khaled (2017). Therapies for advanced stage hepatocellular carcinoma with macrovascular invasion or metastatic disease: A systematic review and meta-analysis. Hepatology.

[4] Yoshida GJ (2016). Emerging role of epithelial-mesenchymal transition in hepatic cancer. J Exp Clin Cancer Res.

[5] van Zijl F, Zulehner G, Petz M, Schneller D, Kornauth C, Hau M, Machat G (2009). Epithelial-mesenchymal transition in hepatocellular carcinoma. Future Oncol.

[6] Lin S, Gregory RI (2015). MicroRNA biogenesis pathways in cancer. Nat Rev Cancer.

[7] Yu J, Lei R, Zhuang X, Li X, Li G, Lev S, Segura MF (2016). MicroRNA-182 targets SMAD7 to potentiate TGFbeta-induced epithelial-mesenchymal transition and metastasis of cancer cells. Nat Commun.

[8] Shi ZM, Wang L, Shen H, Jiang CF, Ge X, Li DM, Wen YY (2017). Downregulation of miR-218 contributes to epithelial-mesenchymal transition and tumor metastasis in lung cancer by targeting slug/ZEB2 signaling. Oncogene.

[9] Tao ZH, Wan JL, Zeng LY, Xie L, Sun HC, Qin LX, Wang L (2013). miR-612 suppresses the invasive-metastatic cascade in hepatocellular carcinoma. J Exp Med.

[10] Sheng L, He P, Yang X, Zhou M, Feng Q (2015). miR-612 negatively regulates colorectal cancer growth and metastasis by targeting AKT2. Cell Death Dis.

[11] Li Y, Tang ZY, Ye SL, Liu YK, Chen J, Xue Q, Chen J (2001). Establishment of cell clones with different metastatic potential from the metastatic hepatocellular carcinoma cell line MHCC97. World J Gastroenterol.

[12] Li Y, Tang Y, Ye L, Liu B, Liu K, Chen J, Xue Q (2003). Establishment of a hepatocellular carcinoma cell line with unique metastatic characteristics through in vivo selection and screening for metastasis-related genes through cDNA microarray. J Cancer Res Clin Oncol.

[13] Wan J, Wen D, Dong L, Tang J, Liu D, Liu Y, Tao Z (2015). Establishment of monoclonal HCC cell lines with organ site-specific tropisms. BMC Cancer.

[14] Langmead B, Salzberg SL (2012). Fast gapped-read alignment with bowtie 2. Nat Methods.

[15] Eisen MB, Spellman PT, Brown PO, Botstein D (1998). Cluster analysis and display of genome-wide expression patterns. Proc Natl Acad Sci U S A.

[16] Saldanha AJ (2004). Java Treeview--extensible visualization of microarray data. Bioinformatics.

[17] Friedlander MR, Mackowiak SD, Li N, Chen W, Rajewsky N (2012). miRDeep2 accurately identifies known and hundreds of novel microRNA genes in seven animal clades. Nucleic Acids Res.

[18] Kim D, Pertea G, Trapnell C, Pimentel H, Kelley R, Salzberg SL (2013). TopHat2: accurate alignment of transcriptomes in the presence of insertions, deletions and gene fusions. Genome Biol.

[19] Anders S, Pyl PT, Huber W (2015). HTSeq--a Python framework to work with high-throughput sequencing data. Bioinformatics.

[20] Yang XR, Xu Y, Yu B, Zhou J, Qiu SJ, Shi GM, Zhang BH (2010). High expression levels of putative hepatic stem/progenitor cell biomarkers related to tumour angiogenesis and poor prognosis of hepatocellular carcinoma. Gut.

[21] Zhu XD, Zhang JB, Zhuang PY, Zhu HG, Zhang W, Xiong YQ, Wu WZ (2008). High expression of macrophage colony-stimulating factor in peritumoral liver tissue is associated with poor survival after curative resection of hepatocellular carcinoma. J Clin Oncol.

[22] Caswell DR, Swanton C (2017). The role of tumour heterogeneity and clonal cooperativity in metastasis, immune evasion and clinical outcome. BMC Med.

[23] Vijapurkar U, Kim MS, Koland JG (2003). Roles of mitogen-activated protein kinase and phosphoinositide 3′-kinase in ErbB2/ErbB3 coreceptor-mediated heregulin signaling. Exp Cell Res.

[24] Ding SZ, Olekhnovich IN, Cover TL, Peek RM, Smith MF, Goldberg JB (2008). Helicobacter pylori and mitogen-activated protein kinases mediate activator protein-1 (AP-1) subcomponent protein expression and DNA-binding activity in gastric epithelial cells. FEMS Immunol Med Microbiol.

[25] Yang X, Liang L, Zhang XF, Jia HL, Qin Y, Zhu XC, Gao XM (2013). MicroRNA-26a suppresses tumor growth and metastasis of human hepatocellular carcinoma by targeting interleukin-6-Stat3 pathway. Hepatology.

[26] Law PT, Qin H, Ching AK, Lai KP, Co NN, He M, Lung RW (2013). Deep sequencing of small RNA transcriptome reveals novel non-coding RNAs in hepatocellular carcinoma. J Hepatol.

[27] Lee H, Shin CH, Kim HR, Choi KH, Kim HH (2017). MicroRNA-296-5p promotes invasiveness through downregulation of nerve growth factor receptor and Caspase-8. Mol Cells.

[28] Lopez-Bertoni H, Lal B, Michelson N, Guerrero-Cazares H, Quinones-Hinojosa A, Li Y, Laterra J (2016). Epigenetic modulation of a miR-296-5p:HMGA1 axis regulates Sox2 expression and glioblastoma stem cells. Oncogene.

[29] Li T, Lu YY, Zhao XD, Guo HQ, Liu CH, Li H, Zhou L (2014). MicroRNA-296-5p increases proliferation in gastric cancer through repression of caudal-related homeobox 1. Oncogene.

[30] Lee KH, Lin FC, Hsu TI, Lin JT, Guo JH, Tsai CH, Lee YC (2014). MicroRNA-296-5p (miR-296-5p) functions as a tumor suppressor in prostate cancer by directly targeting Pin1. Biochim Biophys Acta.

[31] Xu C, Li S, Chen T, Hu H, Ding C, Xu Z, Chen J (2016). miR-296-5p suppresses cell viability by directly targeting PLK1 in non-small cell lung cancer. Oncol Rep.

[32] Maia D, de Carvalho AC, Horst MA, Carvalho AL, Scapulatempo-Neto C, Vettore AL (2015). Expression of miR-296-5p as predictive marker for radiotherapy resistance in early-stage laryngeal carcinoma. J Transl Med.

[33] Vignot S, Lefebvre C, Frampton GM, Meurice G, Yelensky R, Palmer G, Capron F (2015). Comparative analysis of primary tumour and matched metastases in colorectal cancer patients: evaluation of concordance between genomic and transcriptional profiles. Eur J Cancer.

[34] Creighton CJ, Chang JC, Rosen JM (2010). Epithelial-mesenchymal transition (EMT) in tumor-initiating cells and its clinical implications in breast cancer. J Mammary Gland Biol Neoplasia.

[35] Lee SJ, Kim KH, Park KK (2014). Mechanisms of fibrogenesis in liver cirrhosis: the molecular aspects of epithelial-mesenchymal transition. World J Hepatol.

[36] Liu F, Wu L, Wang A, Xu Y, Luo X, Liu X, Hua Y (2017). MicroRNA-138 attenuates epithelial-to-mesenchymal transition by targeting SOX4 in clear cell renal cell carcinoma. Am J Transl Res.

[37] Lin XJ, He CL, Sun T, Duan XJ, Sun Y, Xiong SJ (2017). Hsa-miR-485-5p reverses epithelial to mesenchymal transition and promotes cisplatin-induced cell death by targeting PAK1 in oral tongue squamous cell carcinoma. Int J Mol Med.

[38] Karbownik MS, Szemraj J, Wieteska L, Antczak A, Gorski P, Kowalczyk E, Pietras T (2016). Antipsychotic drugs differentially affect mRNA expression of genes encoding the Neuregulin 1-downstream ErbB4-PI3K pathway. Pharmacology.

[39] Jones MR, Lim H, Shen Y, Pleasance E, Ch'ng C, Reisle C, Leelakumari S (2017). Successful targeting of the NRG1 pathway indicates novel treatment strategy for metastatic cancer. Ann Oncol.

[40] Jeong H, Kim J, Lee Y, Seo JH, Hong SR, Kim A (2014). Neuregulin-1 induces cancer stem cell characteristics in breast cancer cell lines. Oncol Rep.

