# Supplementary material for: miR-296-5p suppresses EMT of hepatocellular carcinoma via attenuating NRG1/ERBB2/ERBB3 signaling
Source: J Exp Clin Cancer Res. 2018 Nov 29;37:294. doi: 10.1186/s13046-018-0957-2 (PMC6264612; doi:10.1186/s13046-018-0957-2)
Supplement: Supplementary file 1 — Table S1. HCC cell lines with different metastatic potentials. (DOCX 20 kb) [file 13046_2018_957_MOESM1_ESM.docx]

**Table S1. HCC cell lines with different metastatic potentials**

| Cell line | chracteristics | Metastatic potential |
| --- | --- | --- |
| MHCC97L  MHCC97H  CSQT-1  CSQT-2  HCCLM3  HCCLM1-S3  HCCLM1-S4  HCCLM1-S5  HCCLM1-S11  HCCLnM1-S11  HCCLnM1-S13  Huh7  Hep3B  SMMC-7721  Bel-7402  PLC/5  HepG2  SNU-398 | HCC clone cells with 40% upon orthotopic inoculation in nude mice  HCC clone cells with 100% lung metastasis in nude mice  Cell line derived from portal vein tumor thrombus of HCC patients Subclonal cell line of CSQT-1 with 100% portal vein tumor thrombus in nude mice  Subclonal cell line of MHCC97H with 100% tumorigenicity, 100% abdominal wall metastases, 100% intrahepatic and pulmonary metastases in nude mice.  Subclonal cell line of HCCLM3 with 100% pulmonary metastases in nude mice  Subclonal cell line of HCCLM3 with 100% pulmonary metastases in nude mice  Subclonal cell line of HCCLM3 with 100% pulmonary metastases in nude mice  Subclonal cell line of HCCLM3 with 75% pulmonary metastases in nude mice.  Subclonal cell line of HCCLM3 with 100% pulmonary and lymphatic metastasis in nude mice  Subclonal cell line of HCCLM3 with 25% pulmonary and lymphatic metastasis in nude mice  Human well differentiated hepatocyte derived cellular cancer  Human HCC  Human HCC with hepatitis, liver cirrhosis  Human HCC with nodular cirrhosis  Human HCC  Human HCC  Human HCC | High  High  High  High  High  High  High  High  High  High  High  Low  Low  Low  Low  Low  Low  Low |

HCC: Hepatocellular carcinoma
