# Supplementary material for: miR-296-5p suppresses EMT of hepatocellular carcinoma via attenuating NRG1/ERBB2/ERBB3 signaling
Source: J Exp Clin Cancer Res. 2018 Nov 29;37:294. doi: 10.1186/s13046-018-0957-2 (PMC6264612; doi:10.1186/s13046-018-0957-2)
Supplement: Supplementary file 2 — Table S2. Sequence of primers for Real-time PCR. (DOC 34 kb) [file 13046_2018_957_MOESM2_ESM.doc]

**Table S2. Sequence of primers for Real-time PCR**

| **Gene** | **Forward primer (5’------3’)** | **Reverse primer(5’------3’)** |
| --- | --- | --- |
| NRG1 | CGGTGTCCATGCCTTCCAT | GTGTCACGAGAAGTAGAGGTCT |
| Fra-1 | CATGACCACACCCTCCCTAACTCC | GTCTCCGCTGCTGCTACTCTT |
| Fra-2 | CAGAAATTCCGGGTAGATATGCC | GGTATGGGTTGGACATGGAGG |
| c-Fos | CCGGGGATAGCCTCTCTTACT | CCAGGTCCGTGCAGAAGTC |
| FosB | GCTGCAAGATCCCCTACGAAG | ACGAAGAAGTGTACGAAGGGTT |
| c-Jun | TCCAAGTGCCGAAAAAGGAAG | CGAGTTCTGAGCTTTCAAGGT |
| JunB | ACGACTCATACACAGCTACGG | GCTCGGTTTCAGGAGTTTGTAGT |
| GAPDH | GGGGCTCTCCAGAACATCATCC | ACGCCTGCTTCACCACCTCTT |
