# Supplementary material for: miR-296-5p suppresses EMT of hepatocellular carcinoma via attenuating NRG1/ERBB2/ERBB3 signaling
Source: J Exp Clin Cancer Res. 2018 Nov 29;37:294. doi: 10.1186/s13046-018-0957-2 (PMC6264612; doi:10.1186/s13046-018-0957-2)
Supplement: Supplementary file 3 — Table S3. Correlations of miR-296-5p expression with the clinicopathological features of HCC. (DOCX 18 kb) [file 13046_2018_957_MOESM3_ESM.docx]

**Table S3. Correlations of miR-296-5p expression with the clinicopathological features of HCC**

| **Clinicopathological Variables** | **n** | **miR-296-5p Expression** | | ***P* value** |
| --- | --- | --- | --- | --- |
|  |  | **Low expression(44)** | **High expression(45)** |  |
| **Sex** |  |  |  |  |
| Male | 71 | 35 | 36 | 0.957 |
| Female | 18 | 9 | 9 |  |
| **Age, years** |  |  |  |  |
| ≤60 | 51 | 24 | 27 | 0.603 |
| >60 | 38 | 20 | 18 |  |
| **HBsAg** |  |  |  |  |
| Negative | 13 | 7 | 6 | 0.731 |
| Positive | 76 | 37 | 39 |  |
| **HBcAb** |  |  |  |  |
| Negative | 11 | 5 | 6 | 0.778 |
| Positive | 78 | 39 | 39 |  |
| **Liver cirrhosis** |  |  |  |  |
| Absence | 23 | 11 | 12 | 0.857 |
| Presence | 66 | 33 | 33 |  |
| **Tumor size, cm** |  |  |  |  |
| ≤5cm | 52 | 19 | 33 | **0.004** |
| ＞5cm | 37 | 25 | 12 |  |
| **AFP,** **ng/mL** |  |  |  |  |
| <20 | 41 | 23 | 18 | 0.246 |
| ≥20 | 48 | 21 | 27 |  |
| **Capsulation formation** |  |  |  |  |
| Absence | 45 | 23 | 22 | 0.750 |
| Presence | 44 | 21 | 23 |  |
| **Microvascular invasion** |  |  |  |  |
| Absence | 39 | 11 | 28 | **＜0.001** |
| Presence | 50 | 33 | 17 |  |
| **Edmondson-Steiner grade** |  |  |  |  |
| I & II | 51 | 14 | 37 | **＜0.001** |
| III & IV | 38 | 30 | 8 |  |
