# Supplementary material for: miR-296-5p suppresses EMT of hepatocellular carcinoma via attenuating NRG1/ERBB2/ERBB3 signaling
Source: J Exp Clin Cancer Res. 2018 Nov 29;37:294. doi: 10.1186/s13046-018-0957-2 (PMC6264612; doi:10.1186/s13046-018-0957-2)
Supplement: Supplementary file 4 — Table S4. Univariate and multivariate analyses of risk factors associated with overall survival of HCC patients. (DOCX 20 kb) [file 13046_2018_957_MOESM4_ESM.docx]

**Table S4. Univariate and multivariate analyses of risk factors associated with overall survival** **of HCC patients**

|  |  | Overall Survival | | | | | |
| --- | --- | --- | --- | --- | --- | --- | --- |
| Variables | n | Univariate analysis | |  | Multivariate analysis | |  |
|  |  | HR(95% CI) | *P* |  | HR(95% CI) | *P* | |
| Sex |  |  |  |  |  |  | |
| Male | 71 | 1 | 0.536 |  |  | NA | |
| Female | 18 | 1.217(0.653-2.267) |  |  |  |  |  |
| Age, years |  |  |  |  |  |  | |
| ≤60 | 51 | 1 | 0.354 |  |  | NA | |
| >60 | 38 | 1.284(0.757-2.177) |  |  |  |  |  |
| HBsAg |  |  |  |  |  |  | |
| Negative | 13 | 1 | 0.570 |  |  | NA | |
| Positive | 76 | 1.259(0.568-2.791) |  |  |  |  |  |
| HBcAb |  |  |  |  |  | NA | |
| Negative | 11 | 1 | 0.876 |  |  |  |  |
| Positive | 78 | 1.066(0.480-2.367) |  |  | NA |  |  |
| Liver cirrhosis |  |  |  |  |  |  | |
| Absence | 23 | 1 | 0.360 |  |  | NA | |
| Presence | 66 | 1.321(0.728-2.399) |  |  |  |  |  |
| Tumor size, cm |  |  |  |  |  |  | |
| ≤5cm | 52 | 1 | **＜0.001** |  | 1 | **0.048** | |
| ＞5cm | 37 | 2.674(1.566-4.567) |  |  | 1.939(1.005-3.739) |  |  |
| AFP, ng/mL |  |  |  |  |  |  | |
| <20 | 41 | 1 | 0.675 |  |  | NA | |
| ≥20 | 48 | 0.894(0.531-1.507) |  |  |  |  |  |
| Capsulation formation formation formation |  |  |  |  |  |  | |
| Absence | 45 | 1 | 0.073 |  |  | NA | |
| Presence | 44 | 0.612 (0.358-1.047) |  |  |  |  |  |
| Microvascular invasion |  |  |  |  |  |  | |
| Absence | 39 | 1 | **＜0.001** |  | 1 | **0.032** | |
| Presence | 50 | 3.040(1.705-5.420) |  |  | 2.025(1.062-3.861) |  |  |
| Edmondson-Steiner grade |  |  |  |  |  |  | |
| I & II | 51 | 1 | **0.002** |  | 1 | NS | |
| III & IV | 38 | 2.309(1.367-3.900) |  |  | 1.054(0.545-2.039) |  |  |
| miR-296-5p expression |  |  |  |  |  |  | |
| Low | 44 | 1 | **0.001** |  | 1 | **0.017** | |
| High | 45 | 0.386(0.225-0.664) |  |  | 0.479(0.263-0.875) |  |  |
