# Supplementary material for: miR-296-5p suppresses EMT of hepatocellular carcinoma via attenuating NRG1/ERBB2/ERBB3 signaling
Source: J Exp Clin Cancer Res. 2018 Nov 29;37:294. doi: 10.1186/s13046-018-0957-2 (PMC6264612; doi:10.1186/s13046-018-0957-2)
Supplement: Supplementary file 5 — Table S5. Univariate and multivariate analyses of risk factors associated with disease-free survival of HCC patients. (DOCX 20 kb) [file 13046_2018_957_MOESM5_ESM.docx]

**Table S5. Univariate and multivariate analyses of risk factors associated with disease-free survival** **of HCC patients**

|  |  | Disease-free survival | | | | | | | | | | |  |  |  |  |
| --- | --- | --- | --- | --- | --- | --- | --- | --- | --- | --- | --- | --- | --- | --- | --- | --- |
| Variables | n | Univariate analysis | | |  | Multivariate analysis | | | | | | | |  |  |  |
|  |  | HR(95% CI) | | *P* |  | HR(95% CI) | | | *P* | | | | |  |  |  |
| Sex |  |  | |  |  |  | | |  | | | | |  |  |  |
| Male | 71 | 1 | | 0.661 |  |  | | | NA | | | | |  |  |  |
| Female | 18 | 0.876(0.484-1.585) | |  |  |  | | |  |  |  |  |  |  |  |  |
| Age, years |  |  | |  |  |  | | |  | | | | |  |  |  |
| ≤60 | 51 | 1 | | 0.197 |  |  | | | NA | | | | |  |  |  |
| >60 | 38 | 1.376(0.848-2.233) | |  |  |  | | |  |  |  |  |  |  |  |  |
| HBsAg |  |  | |  |  |  | | |  | | | | |  |  |  |
| Negative | 13 | 1 | | 0.546 |  |  | | | NA | | | | |  |  |  |
| Positive | 76 | 1.256(0.599-2.635) | |  |  |  | | |  |  |  |  |  |  |  |  |
| HBcAb |  |  | |  |  |  | | |  | | | | |  |  |  |
| Negative | 11 | 1 | | 0.932 |  |  | | | NA | | | | |  |  |  |
| Positive | 78 | 0.968(0.459-2.042) | |  |  |  | | |  |  |  |  |  |  |  |  |
| Liver cirrhosis |  |  | |  |  |  | | |  | | | | |  |  |  |
| Absence | 23 | 1 | | 0.162 |  |  | | | NA | | | | |  |  |  |
| Presence | 66 | 1.494(0.851-2.622) | |  |  |  | | |  |  |  |  |  |  |  |  |
| Tumor size, cm |  |  | |  |  |  | | |  | | | | |  |  |  |
| ≤5cm | 52 | 1 | | **0.002** |  | 1 | | | NS | | | | |  |  |  |
| ＞5cm | 37 | 2.195(1.335-3.608) | |  |  | 1.448(0.788-2.661) | | |  |  |  |  |  |  |  |  |
| AFP, ng/mL |  |  | |  |  |  | | |  | | | | |  |  |  |
| <20 | 41 | 1 | | 0.653 |  |  | | | NA | | | | |  |  |  |
| ≥20 | 48 | 1.895(0.550-1.454) | |  |  |  | | |  |  |  |  |  |  |  |  |
| Capsulation formation formation formation |  |  | |  |  |  | | |  | | | | |  |  |  |
| Absence | 45 | 1 | | 0.528 |  |  | | | NA | | | | |  |  |  |
| Presence | 44 | 0.856(0.529-1.387) | |  |  |  | | |  |  |  |  |  |  |  |  |
| Microvascular invasion |  |  | |  |  |  | | |  | | | | |  |  |  |
| Absence | 39 | 1 | **＜0.001** | |  | 1 | | **0.007** | | | | | | | |  |
| Presence | 50 | 3.048(1.791-5.187) |  | | | | 2.224(1.240-3.988) | | | | |  | | | | |
| Edmondson-Steiner grade |  |  | |  |  |  | | |  | | | | |  |  |  |
| I & II | 51 | 1 | | **＜0.001** |  | 1 | | | | | NS | | | | |  |
| III & IV | 38 | 2.461(1.503-4.029) | |  |  | 1.293(0.698-2.395) | | | | |  |  |  |  |  |  |
| miR-296-5p expression |  |  | |  |  |  | | |  | | | | |  |  |  |
| Low | 44 | 1 | | **＜0.001** |  | 1 | | | | **0.020** | | | | |  |  |
| High | 45 | 0.401(0.244-0.658) | |  |  | 0.523(0.302-0.905) | | | |  |  |  |  |  |  |  |
