# Supplementary figures and images for: miR-296-5p suppresses EMT of hepatocellular carcinoma via attenuating NRG1/ERBB2/ERBB3 signaling
Source: J Exp Clin Cancer Res. 2018 Nov 29;37:294. doi: 10.1186/s13046-018-0957-2 (PMC6264612; doi:10.1186/s13046-018-0957-2)

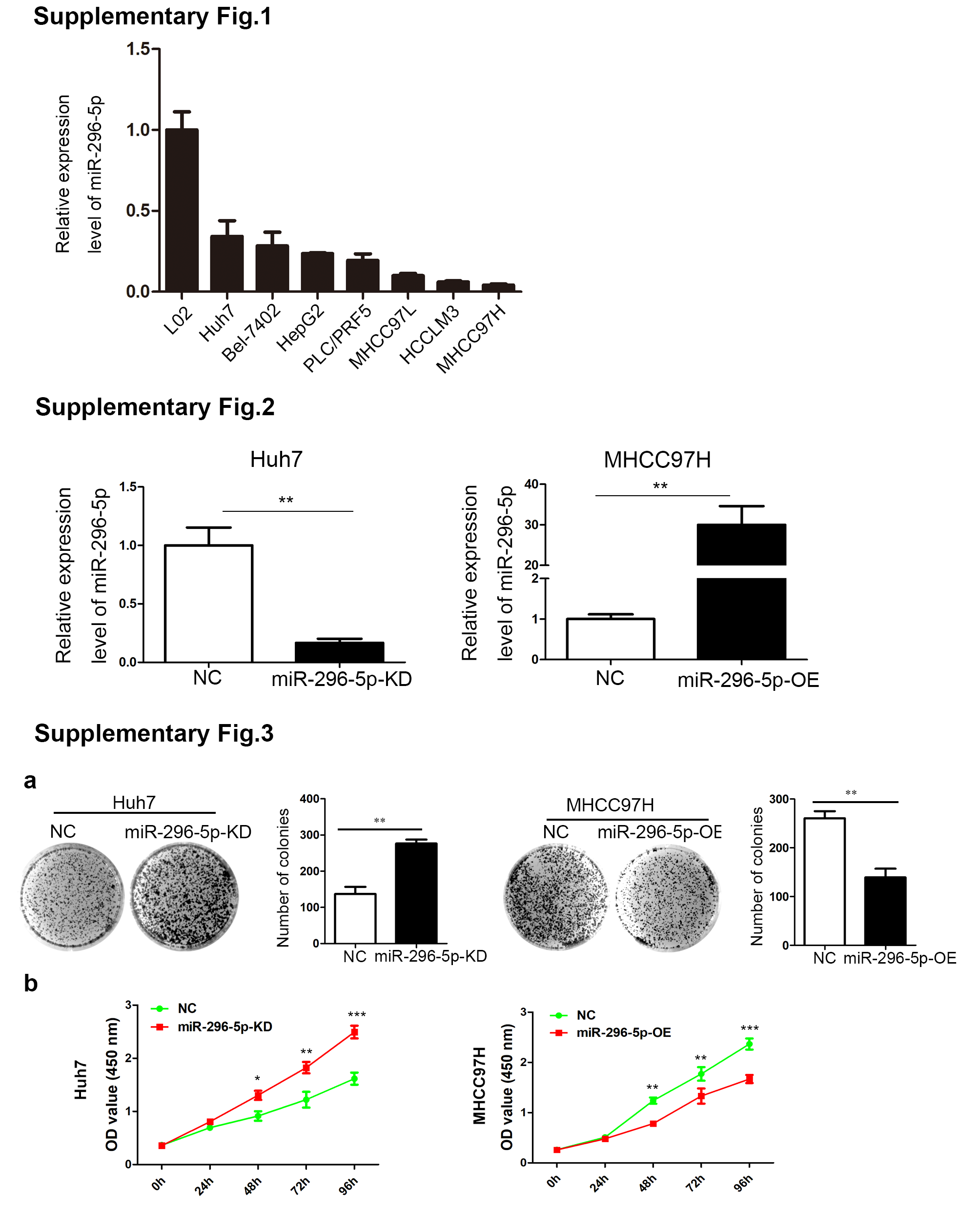

Supplement: Supplementary file 6 — Figure S1. The endogenous levels of miR-296-5p in HCCLM3, MHCC97H, MHCC97L, Huh7 and HepG2 cell lines quantified by qRT-PCR analysis. U6 was used as a control. Figure S2. The miR-296-5p level in Huh7 cells (left) transfected with miR-296-5p knockdown lentiviral vector and MHCC97H cells (right) transfected with miR-296-5p overexpression lentiviral vector analyzed by qRT-PCR. **P < 0.01. Figure S3. The effect of miR-296-5p on HCC cell proliferation. Cell growth capabilities in miR-296-5p-KD Huh7, miR-296-5p-OE MHCC97H cells and their corresponding controls by colony formation (a) and CCK8 assays (b). **P < 0.01. (TIF 621 kb) [file 13046_2018_957_MOESM6_ESM.tif]

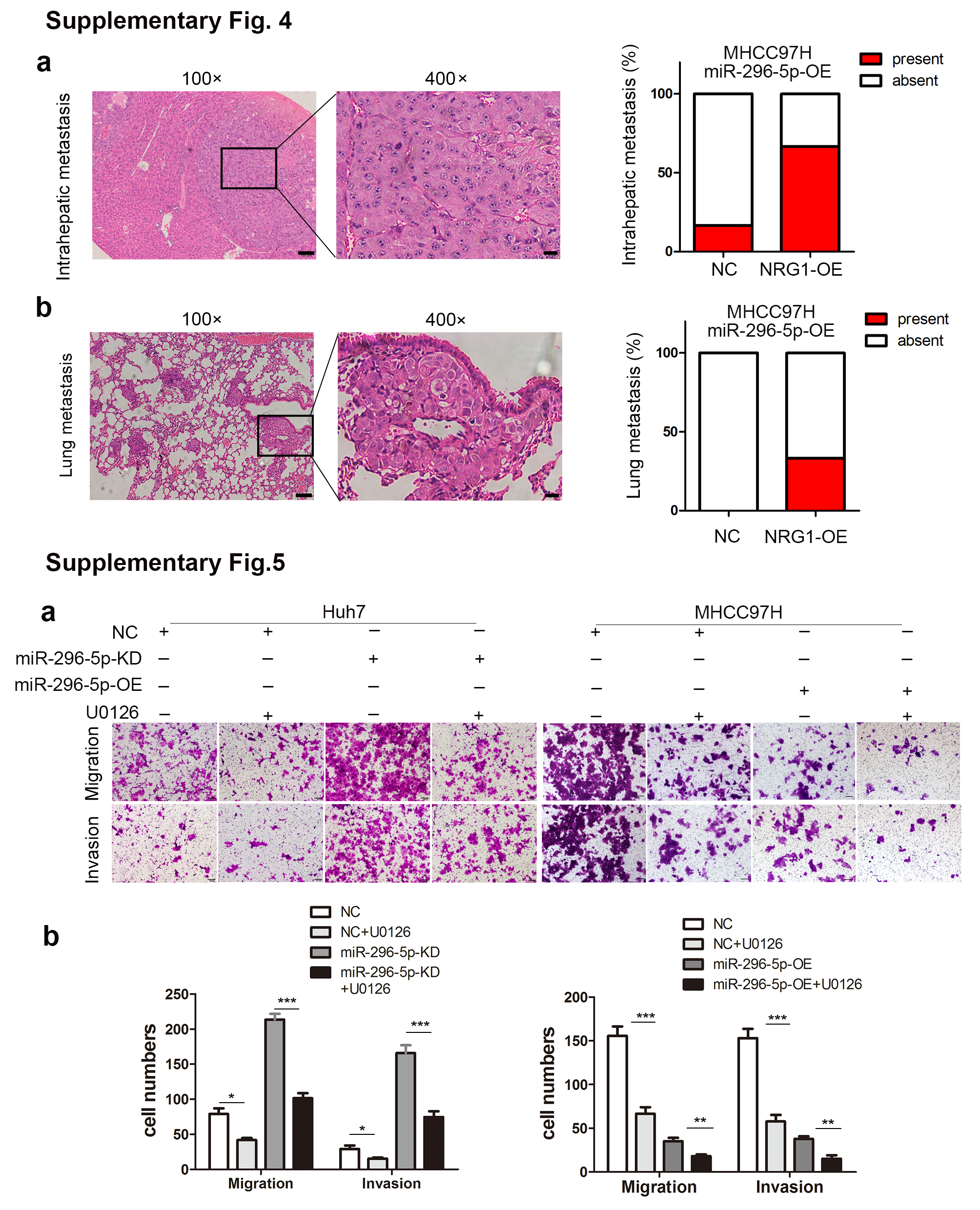

Supplement: Supplementary file 7 — Figure S4. miR-296-5p suppresses in vivo metastasis through NRG1. (a) Hematoxylin and eosin (H&E) staining of metastatic liver nodules (left) and the percentage of mice with or without metastatic nodules in the livers (right). (b) Representative pictures for lung metastasis (left) and the percentage of mice with or without metastatic nodules in the lungs (right). magnification × 100 (left); 400× (right). Figure S5. miR-296-5p mediates HCC cell metastasis through MAPK signaling. (a and b) The cellular invasive and migratory capability in miR-296-5p-KD Huh7, miR-296-5p-OE MHCC97H cells and their corresponding control cells after U0126 treatment. *p < 0.05, **p < 0.01, ***p < 0.001. (TIF 4105 kb) [file 13046_2018_957_MOESM7_ESM.tif]
